# Pervasive relaxed selection in termite genomes

**DOI:** 10.1098/rspb.2023.2439

**Published:** 2024-05-22

**Authors:** Kyle M. Ewart, Simon Y. W. Ho, Al-Aabid Chowdhury, Frederick R. Jaya, Yukihiro Kinjo, Juno Bennett, Thomas Bourguignon, Harley A. Rose, Nathan Lo

**Affiliations:** ^1^ School of Life and Environmental Sciences, University of Sydney, Sydney, New South Wales, Australia; ^2^ Ecology & Evolution, Research School of Biology, Australian National University, Acton, Australian Capital Territory, Australia; ^3^ Okinawa Institute of Science and Technology Graduate University, Okinawa, Japan; ^4^ Okinawa International University, Okinawa, Japan

**Keywords:** Blattodea, effective population size, eusociality, indirect selection, phylogenomics, relaxed selection

## Abstract

Genetic changes that enabled the evolution of eusociality have long captivated biologists. More recently, attention has focussed on the consequences of eusociality on genome evolution. Studies have reported higher molecular evolutionary rates in eusocial hymenopteran insects compared with their solitary relatives. To investigate the genomic consequences of eusociality in termites, we analysed nine genomes, including newly sequenced genomes from three non-eusocial cockroaches. Using a phylogenomic approach, we found that termite genomes have experienced lower rates of synonymous substitutions than those of cockroaches, possibly as a result of longer generation times. We identified higher rates of non-synonymous substitutions in termite genomes than in cockroach genomes, and identified pervasive relaxed selection in the former (24–31% of the genes analysed) compared with the latter (2–4%). We infer that this is due to reductions in effective population size, rather than gene-specific effects (e.g. indirect selection of caste-biased genes). We found no obvious signature of increased genetic load in termites, and postulate efficient purging of deleterious alleles at the colony level. Additionally, we identified genomic adaptations that may underpin caste differentiation, such as genes involved in post-translational modifications. Our results provide insights into the evolution of termites and the genomic consequences of eusociality more broadly.

## 1. Introduction

Eusocial insects represent only approximately 2% of all insect species but have an enormous ecological impact, representing over half of all insect biomass [1]. Eusocial insect colonies comprise highly specialized castes, whereby some members of the colony reproduce while the workers and soldiers focus on tasks such as foraging, defence, brood care and nest construction [2]. These considerably different behaviours arise from the same genome. The behavioural and genetic changes that enabled the evolution of eusociality, and that have facilitated the ecological dominance of these insects, have long interested biologists.

Many studies on the evolution of eusociality have focussed on the genomic features that underpin this trait. However, in recent years, the consequences of eusociality on genome evolution have also become the subject of active research [3–6]. A key parameter of interest is the molecular evolutionary rates of eusocial organisms compared with their solitary relatives [7,8]. Two main hypotheses have been put forward in relation to the consequences of eusociality on evolutionary rates. The first hypothesis posits that the presence of very few reproductive individuals within colonies should result in eusocial insects having small effective population sizes (*N*_e_). This would be expected to increase the strength of genetic drift in eusocial lineages, leading to higher evolutionary rates (e.g. through weaker purifying selection) [3,5,9–12] (but see [13]). The second hypothesis states that the specific expression of genes in different castes and the reduction of multi-caste pleiotropy should result in distinct selection pressures acting on these genes for the different castes (i.e. ‘indirect selection’), leading to the reduced efficacy of selection [6,7,14]. For example, selection on worker-biased genes is largely indirect in reproductive individuals, such that selection may be weaker in worker-biased genes than in queen-biased genes. Moreover, genes under relaxed selection in eusocial ancestors might more readily undergo caste-biased expression. Under this scenario, genes that are caste-specific in eusocial species might have elevated evolutionary rates in both eusocial and non-eusocial taxa [8].

Most studies on the consequences of eusociality on genome evolution have focussed on hymenopterans, with relatively little attention given to other eusocial insects. These studies have identified elevated rates of genome-wide evolution [3], as well as elevated rates of evolution in differentially expressed caste-specific genes [7,8], supporting both of the aforementioned hypotheses. However, the relative contributions of reduced *N*_e_ and differential gene expression to these elevated rates remain unclear. Further, there has been limited use of phylogenomic approaches to investigate rates of genomic evolution in eusocial insects and their relatives, despite the availability of methods for examining differing molecular evolutionary forces across large numbers of loci. Phylogenomic methods can help to elucidate the timing of shifts in genomic rates, characterize signatures of selection associated with rate changes and identify the specific genes affected.

An important eusocial insect group with significant ecological impacts is the termites. Their evolution of eusociality from cockroach ancestors [15–19], and their diploid chromosome make-up (in contrast to the haplodiploid eusocial species in Hymenoptera), makes them a valuable comparative model system. However, past examinations of genome-wide molecular evolution in termites have typically been based on a single termite representative [3,5], or have lacked comparisons with cockroach relatives [6]. Although a number of termite genomes have been sequenced [20–23], genomes are available for only two cockroach species, the pests *Periplaneta americana* (Blattidae [24]) and *Blattella germanica* (Ectobiidae [21]). A lack of genomic resources hinders our ability to perform in-depth comparisons of termites and cockroaches to examine the consequences of eusociality on genome evolution.

Here, we facilitate comparative phylogenomic analyses of Blattodea by sequencing the genomes of three Australian species from Blaberidae, the most speciose cockroach family. *Panesthia cribrata* (subfamily Panesthiinae) nests in and feeds on rotting wood, but displays minimal social behaviour. *Geoscapheus dilatatus* and *Neogeoscapheus hanni* (subfamily Geoscapheinae) feed on dried leaf litter and form burrows in the soil up to a metre deep, in which offspring remain with their mothers for several months. The inclusion of *P. cribrata* in our analyses allowed us to control for the effects of wood nesting and wood feeding on genome evolution, in the absence of eusocial behaviour.

We use these new data in conjunction with previously generated genomes from Blattodea to achieve three main objectives: (1) increase our understanding of patterns of selection across termite and cockroach genomes; (2) to evaluate factors that have been posited to influence molecular evolutionary rates in eusocial organisms, such as a reduced *N*_e_ and indirect selection of caste-biased genes; and (3) to identify genomic adaptations that underpin the evolution of eusociality in termites. We expect that this study will shed light on the evolution of termites and eusociality more broadly.

## 2. Results and discussion

### (a) Genome assemblies and orthologue assignment

We produced novel de novo genome assemblies for *G. dilatatus*, *N. hanni* and *P. cribrata* using a combination of high-throughput sequencing approaches (electronic supplementary material, table S1; figure S1). The genome quality for *G. dilatatus* was comparable to that of the well-studied pest cockroach species *Periplaneta americana* and *Blattella germanica* (electronic supplementary material, table S2 [21,24]), while genome completeness and continuity for *P. cribrata* and *N. hanni* were lower (table 1). Annotated protein-coding genes were BLASTed against the insect protein database, resulting in an average sequence identity of greater than 65% for each species (see electronic supplementary material, figure S2 for annotation QC results). Most annotated genes (91.7%) were assigned to orthogroups, and relatively few were found to be species-specific (electronic supplementary material, figure S3). Of the 24 455 orthogroups, 1491 were single-copy and present in all species; we used these single-copy orthologues in the subsequent evolutionary analyses.

**Table 1.** Genome assembly and annotation metrics. Ab initio predictions that had no BLAST hits were not included in the numbers of annotated genes.

species	genome length (Gb)	N50	no. of contigs	complete BUSCOs (%)	fragmented BUSCOs (%)	missing BUSCOs (%)	duplicated BUSCOs (%)	no. of annotated genes
*G. dilatatus*	1.70	3.2 Mb	84 284	93.9	4.8	1.3	0.7	30 006
*N. hanni*	1.68	11.4 kb	270 019	69.0	19.5	11.5	0.7	38 314
*P. cribrata*	1.78	298 kb	204 297	85.0	10.9	4.1	1.8	39 852

### (b) Pervasive relaxed selection in termite genomes

By investigating patterns of non-synonymous (*d*_N_) and synonymous (*d*_S_) changes, we found evidence of pervasive relaxed selection on the termite terminal branches of the phylogeny. Our analyses included estimates of *d*_N_ and *d*_S_ for all single-copy orthologues in nine examined taxa: the three newly sequenced cockroach species (*Geoscapheus dilatatus*, *Neogeoscapheus hanni* and *Panesthia cribrata*), two pest cockroach species (*Periplaneta americana* and *Blattella germanica*), three termite species (*Cryptotermes secundus*, *Coptotermes formosanus* and *Zootermopsis nevadensis*), and one outgroup species (the orthopteran *Laupala kohalensis*).

We applied the free-ratio model in CODEML [25], allowing a separate *d*_N_ and *d*_S_ on each branch, and found that median *d*_N_ rates were elevated on the termite branches (excluding the termite stem; median = 4.19 × 10^−4^) compared with the cockroach branches (excluding the Blattodea stem; median = 2.85 × 10^−4^). Conversely, the *d*_S_ rate was found to be lower on the termite branches (median = 2.66 × 10^−3^) than on the cockroach branches (median = 3.46 × 10^−3^). The median *d*_N_/*d*_S_ for the termite branches was 0.149, while that for cockroach branches was 0.081. This median termite *d*_N_/*d*_S_ is considerably higher than previously reported estimates for eusocial hymenopteran species based on genomic data such as bees (*Apis*, *Bombus*, and *Tetragonula* spp.; average corrected *d*_N_/*d*_S_ = 0.109 [12]; a separate group of Apidae spp.; median *d*_N_/*d*_S_ = 0.081 [26]), ants (comprising Formicidae spp.; average corrected *d*_N_/*d*_S_ = 0.107 [12]) and wasps (comprising Polistinae and Vespinae spp.; average corrected *d*_N_/*d*_S_ = 0.103 [12]), while the median cockroach *d*_N_/*d*_S_ falls within the range of estimates for solitary hymenopteran species [12,26]. However, caution should be taken when directly comparing *d*_N_/*d*_S_ estimates across studies based on different sets of genes with varying levels of conservation, and which have employed different models/methods to estimate *d*_N_/*d*_S_.

**Figure 1.**
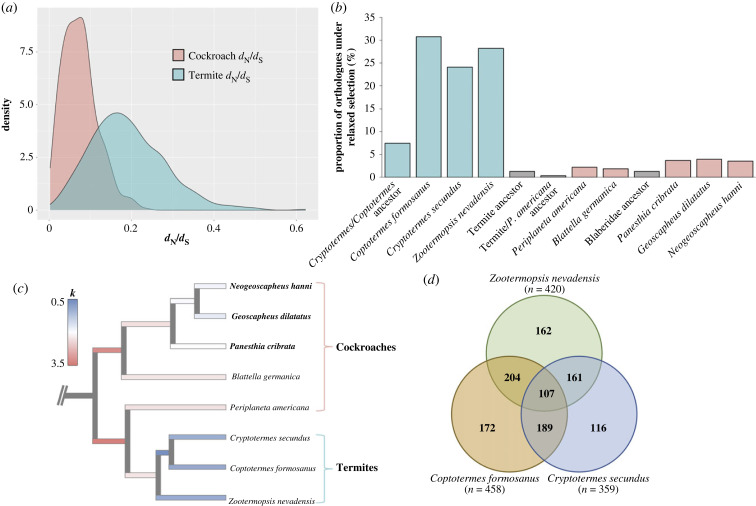
(*a*) Density plot for the three-ratio *d*_N_/*d*_S_ model for the cockroaches and termites. (*b*) Proportion of genes under significant relaxed selection for each of the focal branches based on the RELAX analyses (after Holm–Bonferroni correction). (*c*) Phylogenetic tree of analysed termite and cockroach genomes (genomes generated in this study in bold), with branches coloured by their median relaxed selection parameter (*k*) determined using RELAX, whereby *k* < 1 indicates relaxed selection and *k* > 1 indicates intensified selection (the outgroup has been pruned from the tree). (*d*) Venn diagram displaying overlapping orthologues under relaxed selection on the termite terminal branches.

Separate analyses of *d*_N_/*d*_S_ utilizing a three-ratio model (allowing a separate ratio for the termite clade) was preferred over a two-ratio model (allowing one ratio for the outgroup and one for the entire cockroach–termite ingroup) for 75.7% of the analysed orthologues (*p* < 0.05; 45.1% after applying a Holm–Bonferroni correction). The distribution of *d*_N_/*d*_S_ on termite branches in this analysis was wider and had a much higher mean than that for the cockroach branches (figure 1*a*). In 90.8% of the analysed orthologues, the termite *d*_N_/*d*_S_ was higher than the cockroach *d*_N_/*d*_S_ (significantly higher for 73% when applying *p* < 0.05, and 44.5% after applying a Holm–Bonferroni correction).

To investigate further the differences in *d*_N_, *d*_S_ and *d*_N_/*d*_S_ between termite and cockroach branches, we tested for relaxed selection using the ‘RELAX’ method [27], implemented in HyPhy [28,29]. We detected a large proportion of the single-copy orthologues under relaxed selection on the termite terminal branches (24.09–30.74%) compared with the cockroach terminal branches (1.88–3.96%) (after a Holm–Bonferroni correction for testing multiple branches; figure 1*b*). When we analysed an audited subset of single-copy orthologues (i.e. gene alignments that were manually inspected and edited if required), we obtained similar proportions (electronic supplementary material, table S3). This scale of relaxed selection is comparable to inferences from a previous study on hymenopteran eusocial species [12], which identified 23.21% (after a conservative Bonferroni correction) of the analysed orthologues to be under relaxed selection (though direct comparison between these studies should be undertaken with caution). A separate study analysed relatively small sets of genes (13–78 single-copy orthologues [30]) in termites, and found 18–27% of the genes to be under relaxed selection in four of the termite species analysed, including the three termite genera analysed in our study. However, for the other four termite species analysed, lower proportions (0–12%) of the genes were identified as being under relaxed selection (comparisons with cockroaches were not included). The difference in relaxed selection between termites and cockroaches in our analyses becomes more extreme when analysing all termite branches together (72.43% of orthologues analysed) compared with all cockroach branches (0.74%). In relaxed selection analyses, a significant result of *k* < 1 is indicative of a reduction in selection strength, while *k* > 1 indicates an intensification of selection strength. Median *k* values were found to be the lowest on termite branches, and well below one (figure 1*c*).

Given that the three termite species in our study form a single monophyletic clade, we note the issue of phylogenetic non-independence in some of our analyses. However, we do not believe that this affects the key findings outlined above. When analysing individual branches, a large proportion of the genes were under relaxed selection in only one or two of the termite terminal branches (figure 1*d*). Hence, although reductions in *N*_e_ are likely to have occurred in the ancestral termite lineage following its emergence approximately 134 mya [19], the genomic consequences of being eusocial are likely to have manifested independently in each of the three analysed termite species since they diverged from one another approximately 97–107 mya [19].

Our analyses investigated a relatively conserved set of single-copy orthologues present among the highly divergent taxa included. The scale of *d*_N_/*d*_S_ and relaxed selection likely differs across genes that are faster evolving, species-specific, and/or part of multigene families. We expect that the patterns and causes of relaxed selection in such genes would be similar to those that we have analysed (see below).

### (c) Relaxed selection in termite genomes is better explained by reduced *N*_e_ rather than indirect selection

We evaluated two main scenarios that may explain the pervasive selection in termite genomes: (1) a reduction in *N*_e_, and (2) indirect selection. A reduction in *N*_e_ would have a genome-wide effect, whereby the strength of genetic drift increases for every gene. Conversely, indirect selection would have gene-specific effects. Only caste-specific genes are expected to experience reduced selective constraints, as they are only expressed in a subset of the colony (e.g. worker-biased genes are predominantly expressed by workers, not reproductives). We note that a niche- or functional-specialization scenario would also confer gene-specific effects (elaborated in section ‘d’ below).

To investigate whether indirect selection underpinned signatures of relaxed selection in our data set, we identified and analysed 311 genes in our single-copy orthologue data set that had previously been identified to display either unbiased or caste-biased differential expression [6,31] (noting that caste-biased genes in *Coptotermes formosanus* were not identified in these studies). We found no significant difference between the proportion of caste-biased (33.3% for *Zootermopsis nevadensis* and 24.4% for *Cryptotermes secundus*) and unbiased (25.3% for *Z. nevadensis* and 20% for *C. secundus*) orthogroups under relaxed selection when compared with proportions in the full single-copy orthologue data set (chi-squared tests, *p* = 0.29 for *Zootermopsis nevadensis* and *p* = 1 for *Cryptotermes secundus* caste-biased genes; electronic supplementary material, table S4). Similar patterns were found for genes only expressed in one caste (electronic supplementary material, table S4). Further, if indirect selection were the main driver of these patterns of relaxed selection, we would expect to see considerably fewer genes affected. Previous RNAseq analyses showed that many caste-biased genes are species specific [6] and/or part of multi-gene families [31]. Such genes were not considered in our single-copy orthologue data set and require further investigation.

The pervasive scale of relaxed selection across the termite genomes is explained better by a reduction in *N*_e_ than by gene-specific effects resulting from indirect selection. A reduction in *N*_e_ increases the intensity of genetic drift [32], and consequently reduces the efficacy of both negative and positive selection [27,33]. Termites are expected to have a very low *N*_e_ relative to their census population size due to the complex social structure of their colonies, the small number of reproductive individuals (typically one king and one queen per colony), and their history of inbreeding within colonies [11,34,35]. Indeed, Romiguier *et al*. [3] found that the termite *Reticulitermes grassei* had a large reduction in *N*_e_, even compared with other eusocial species, and postulated that this was due to the strict monogamy seen in this taxon.

Although a reduced *N*_e_ will theoretically not directly affect the substitution rate at neutral sites [36], a long-term low *N*_e_ has been proposed to lead to the evolution of a higher mutation rate [37]. However, we found that *d*_S_ values were lower in termites than in cockroaches. This result can be explained by the considerably longer generation time of termites (5–10 years) compared with cockroaches (typically less than 1 year). Although a longer generation time is expected to increase the mutation rate per generation (due to increased cell divisions of gametes, and greater time for DNA damage to accumulate), the per-year mutation rate is expected to decrease [38,39]. However, a reduction of *N*_e_ appears to have counteracted this generation-time effect for non-neutral sites in termites (based on observed *d*_N_ rates).

### (d) Alternative explanations for relaxed selection in termite genomes

Although reduced *N*_e_ is the most likely explanation for the pervasive relaxed selection observed in our study, we are unable to rule out the possibility that indirect selection or other factors contributed to relaxed selection in at least some of the examined orthologues. Although many genes were only under significant relaxed selection on one of the three termite branches (figure 1*d*), there were more genes under relaxed selection that overlap in all three termite species (*n* = 107) than expected by chance. Hence, some ‘core genes’ might be under relaxed selection due to other non-random processes, such as indirect selection and/or reduced functional importance in termites. There was no evidence that caste-biased genes were undergoing relaxed selection or accelerated evolution prior to becoming caste-biased, as alluded to by previous authors [8,40,41], based on evolutionary rates of the cockroach orthologues (electronic supplementary material, figures S4, S5) and the minimal relaxed selection on the termite ancestral branch (figure 1*b,c*).

The intensity of selection is proportional to the product of *N*_e_ and the selection coefficient *s* [36]. Therefore, it is possible to observe pervasive relaxed selection without a reduction in *N*_e_, given a change in *s* that affects many loci. Previous authors have hypothesized that the cooperative interactions of social species can buffer or compensate for the expression of deleterious mutations, particularly in relation to immune responses [30,42–46]. The creation of a benign environment through social cooperation (e.g. termite mounds) and/or buffering for the expression of deleterious alleles (e.g. through food allocation) will lead to a reduction in selection pressures for the genes involved [46]. For example, termite social hygiene behaviours, such as allogrooming, might result in relaxation of selection in certain innate components of their immune system [45].

**Figure 2.**
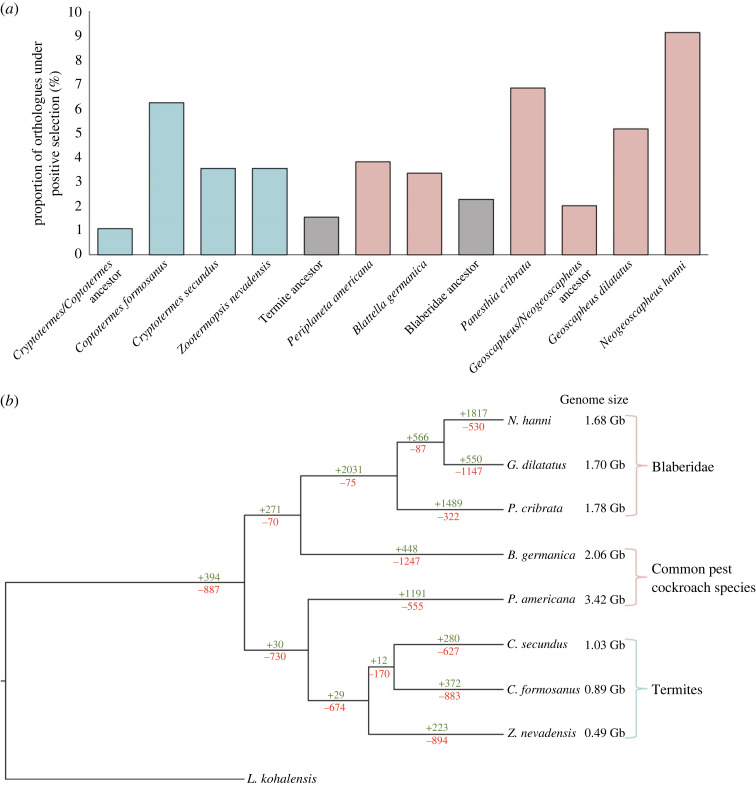
(*a*) Proportion of orthologues (*n* = 1491) on various branches undergoing positive selection, determined using aBSREL analyses (see electronic supplementary material, table S6, for names of genes undergoing positive selection in termite taxa). (*b*) Number of gene expansions (green) and contractions (red) on each branch of the cockroach/termite tree (see electronic supplementary material, table S7, for names of genes that had significant expansions/contractions in termite taxa). The number of gene expansions/contractions combined results from the small and large orthogroups analysed.

Although there might have been large-scale shifts in *s* across termite genomes since divergence from their subsocial cockroach ancestors, we have several reasons to believe that this is not the primary driver of the patterns of relaxed selection observed. First, said patterns do not appear to be associated with specific habitats. The different termite species analysed occupy different niches: *Coptotermes formosanus* constructs nests and forages in the soil; *Zootermopsis nevadensis* lives and feeds in dead dampwood; and *Cryptotermes secundus* lives and feeds in dead drywood. Likewise, the five sampled cockroach species live in varying habitats, from urban environments to wood and soil burrows. Hence, if pervasive relaxed selection were caused by a reduced *s* due to differing selective pressures across different niches (and not a reduction in *N*_e_), we might expect relaxed selection to be restricted to one or two of the termite species, or observe comparable levels of relaxed selection in some cockroach species (e.g. *Periplaneta americana*, a recently urbanized cockroach species, sister lineage to the termite species in our analysis). Second, if a reduced *s* across many genes were due to the social benefits of group living (e.g. allogrooming), we might expect a larger overlap of orthologues to be under relaxed selection (figure 1*d*; but see [30]), and a larger proportion of orthologues under relaxed selection in the termite ancestral branches. Third, given the relatively conserved data set that we analysed (i.e. single-copy orthologues across taxa approximately 350 million years divergent [47]), we expect most genes to be functionally important in termites (i.e. a relatively stable *s* across the analysed phylogeny). Hence, a reduction in *s* across greater than 24% of the relatively conserved orthologue data set is unlikely. Fourth, if some of the analysed orthologues were indeed associated with changes in niche or function, we might also expect to see signatures of positive selection in termites; however, this was not the case (figure 2*a*). Therefore, although we cannot rule out a pervasive reduction of *s* across the termite genome due to increased social cooperation or niche specialization, or a combination of reduced *s* and indirect selection, our results suggest that the observed patterns of relaxed selection are dominated by the genome-wide effects of a reduced *N*_e_, rather than gene-specific effects.

### (e) No obvious signature of increased genetic load in termites

A long-term small *N*_e_ and pervasive relaxed selection could result in an accumulation and fixation of deleterious alleles and a high genetic load [3,33,48,49]. Unexpectedly, however, termites do not appear to have a higher genetic load than cockroaches based on the number of derived putative deleterious substitutions (identified using PROVEAN [50]) in 581 Insecta BUSCOs (i.e. highly conserved genes). Here, we are referring only to ‘realized load’ and not ‘masked load’ [49,51], given that we are comparing amino acid sequences across an order of insects, and not considering heterozygosity. Termites had a higher number of total substitutions per amino acid than the cockroach species (significantly higher for 12/15 pairwise comparisons, and non-significantly higher for 3/15 pairwise comparisons; electronic supplementary material, figure S6; table S5). However, the number of inferred deleterious substitutions per amino acid was significantly lower or similar in termites compared with the cockroach species.

Therefore, despite the patterns of pervasive relaxed selection that we identified, there does not appear to be an obvious signature of increased genetic load in termites compared with cockroaches. How then have termites, an ancient and evolutionarily successful group, succeeded in diversifying and adapting to changing environments? An increased genetic load could have been alleviated through purging, which involves the removal of recessive deleterious alleles exposed by inbreeding. Purging could be particularly effective in termites due to a long-term history of inbreeding and variation in heterozygosity generated by inbreeding [52,53]. Moreover, termites could have experienced purging at the colony level. The survival of dispersing reproductive individuals that attempt to form a new colony relies on the survival of their offspring; colony survival hinges on the worker offspring gathering resources, building the nest structure, and raising offspring. Therefore, if we consider the colony as the basic unit of the population rather than individual termites (as in [34,54–56]), when primary (alate-derived) reproductives disperse and breed, purging could occur by the failure of colonies established by unfit offspring. Indeed, there is evidence that closely related primary reproductives establish colonies less frequently than non-related primary reproductives as a result of inbreeding depression [57] (but see [58–61]). This hypothesis requires further investigation.

The lack of an obvious increased genetic load in termites may also be due to the insensitivity of the methods we applied, particularly given the complexities of delineating benign and deleterious substitutions. Further investigation into the functional effects of mutations in coding regions (e.g. experimental evidence) may help to characterize the extent to which derived mutations are deleterious, and clarify any differences in genetic load in these species [51]. Further, we reiterate issues of phylogenetic non-independence in our data set, though we believe that this has a negligible effect on these genetic load inferences (detailed in the electronic supplementary material).

### (f) Positive selection and gene expansions/reductions in termite and cockroach genomes

In our analyses of positive selection that allowed a separate *d*_N_/*d*_S_ on terminal branches leading to cockroach and termite taxa, we did not detect substantial positive selection in termite genomes (detailed further in the electronic supplementary material). The number of orthologues under positive selection varied between 1.1% and 9.1% across the terminal branches (figure 2*a*), with the clade comprising *P. cribrata*, *G. dilatatus*, and *N. hanni* displaying the highest number. Further, no significant differences were found when comparing the proportions of genes under positive selection that had previously been demonstrated to be unbiased or differentially expressed between castes (electronic supplementary material, table S4).

Previous studies on eusocial species have highlighted the importance of epigenetic mechanisms, particularly DNA methylation, for controlling caste-specific gene expression [21,56,62–67]. Of the 8538 orthogroups that we identified to be displaying gene family size changes (figure 2*b*; detailed further in the electronic supplementary material), numerous expanding gene families in the termite lineages were related to protein ubiquitination (electronic supplementary material, table S7; e.g. E3 ubiquitin-protein ligase, and Ubiquitin carboxyl-terminal hydrolase 45), an important post-translational mechanism, as well as other gene families involved in gene expression regulation (e.g. Histone H4 transcription factor and KAT8 regulatory NSL complex subunit 3). Protein ubiquitination results in protein degradation or modification. Histones are often ubiquitinated, which subsequently alters chromatin structure and gene function, and may therefore influence patterns of DNA methylation [68,69]. Previous studies have demonstrated that these post-translational modifications and histone structure changes influence the development of castes in the honey bee [70–72] and carpenter ant [73,74]. Evidently, various gene expression mechanisms beyond DNA methylation, such as ubiquitination, could be fundamental to the development of castes, and therefore warrant further research.

## 3. Concluding remarks

Incorporating additional genome resources has proven valuable in our comparative genomic analyses of termites and cockroaches. Sequencing and analysing genomes from the sister group of termites, representatives of the wood-feeding genus *Cryptocercus*, will help to elucidate the ancestral signals of the relaxed selection that we have identified, and provide insights into the evolutionary origins of caste-biased genes. Further, given that the termites represent only a single transition to eusociality, expanding the analyses to include genomes of other eusocial groups (e.g. eusocial Hymenoptera, rodents and shrimp) could provide insight into convergent patterns of eusocial evolution (e.g. [75]).

Investigating termite populations with varying *N*_e_ will help to define the relationship between the pervasive relaxed selection identified and the group's demography. Population genetic analyses will also enable categorization of realized and masked genetic load to clarify whether termites exhibit purging of deleterious alleles, and whether this occurs at the colony level. In addition, characterizing selection coefficients of various genes will help to tease apart genome-wide and gene-specific consequences of evolving eusociality, shedding light on patterns of relaxed selection.

Our findings corroborate several previous studies that suggested an association between eusociality and a reduced *N*_e_, resulting in relaxed selection [3,5,12,38]. Although accurate estimation of *N*_e_ is particularly challenging for eusocial species [76], integrating such estimates, or relevant proxies (e.g. [5]), could help to clarify the patterns of relaxed selection that we have inferred, and the association between *N*_e_ and the evolution of eusociality. This, in turn, would help to illuminate the extent to which shifts in *N*_e_ and the emergence of eusociality have shaped genomic evolution in termites.

## 4. Material and methods

### (a) Sample collection and DNA extraction

Samples of *P. cribrata* were collected from North Manly, NSW, in August 2020 by N. Lo. Samples of *G. dilatatus* were collected from Gilgandra, NSW, in September 2019 by H. A. Rose. Samples of *N. hanni* were collected from Mt Molloy, QLD, in January 2020 by J. A. Walker and N. Lo. Muscle tissue was removed from the leg of one adult female of each species for DNA extraction. DNA was extracted using a phenol/chloroform extraction protocol (detailed in the electronic supplementary material).

### (b) DNA sequencing approaches

Three different DNA sequencing approaches were employed to generate the cockroach genomes: linked-read sequencing, chromosomal conformation capture sequencing, and long-read sequencing (electronic supplementary material, table S1). We used two technologies for linked-read sequencing: Transposase Enzyme-Linked Long-read Sequencing (TELL-Seq™ [77]) and single-tube long fragment read (stLFR [78]). These technologies are based on co-barcoding short-read sequences that derive from the same DNA fragment, providing long-range information to enable assembly of long DNA regions.

We used Hi-C sequencing [79], a chromosomal conformation capture sequencing method based on proximity ligation. Hi-C sequencing supports scaffolding of genomic regions that are in close spatial proximity. Hi-C library preparation was performed using the Arima Hi-C Plus kit (Arima, USA) following the manufacturer's protocol for large animal tissue, using the *DpnII* and *HinfI* enzymes. Preliminary sequencing and subsequent analysis using qc3C [80] was performed to estimate the quantity of Hi-C sequencing required.

We utilized the Pacific Biosciences (PacBio) HiFi long-read sequencing technology [81], which uses circular consensus sequencing to generate long-reads that are greater than 99% accurate. The library preparation was performed using the SMRTbell Express Template Prep Kit 2.0 following the manufacturer's instructions (Pacific Biosciences). Additional information on the TELL-Seq, stLFR, Hi-C and PacBio HiFi library preparations can be found in the electronic supplementary material.

### (c) Genome assembly and annotation

Different genome assembly pipelines were utilized for the different species depending on the sequencing resources available (electronic supplementary material, figure S1). The first step for all three species was a de novo assembly based on linked-read data, performed using the Supernova assembler v. 2.1.1 [82]. For the genome of *P. cribrata*, we performed a separate de novo assembly based on the PacBio Hifi sequencing data using Improved Phased Assembler (IPA) v. 1.3.1 (Pacific Biosciences, 2022), then merged this long-read assembly with the linked-read assembly using *quickmerge* [83]. For the genome of *G. dilatatus*, gaps in the Supernova de novo assembly were filled with low-coverage PacBio Hifi data using TGS-GapCloser v. 1.1.1 [84], then the assembly was scaffolded with Hi-C sequence data using SALSA2 [85,86]. All three genome assemblies were annotated using FGENESH++ v. 7.2.2 [87,88], carried out on the Nimbus cloud service (provided by the Pawsey Supercomputing Centre). We generated various statistics to assess assembly continuity using QUAST v. 4.3 [89], and assessed assembly completeness using BUSCO v. 4.0.6 (utilizing the Insecta lineage data set [90–93]). Additional information on genome assembly and annotation methods can be found in the electronic supplementary material.

### (d) Orthology assignment

Proteomes of these three newly generated blaberid annotated genomes were combined with proteomes from six other species extracted from InsectBase 2.0 [94], including: American cockroach (*Periplaneta americana*), German cockroach (*Blatella germanica*), Nevada termite (*Zootermopsis nevadensis*), Formosan termite (*Coptotermes formosanus*), a drywood termite (*Cryptotermes secundus*) and a cricket (*Laupala kohalensis*) as an outgroup (electronic supplementary material, table S2). The cricket proteome was utilized as an outgroup rather than a proteome from a more closely related species (e.g. *Clitarchus hookeri*) due to its relatively high genome completeness. The longest isoform for each gene was extracted using the primary_transcript.py script supplied by OrthoFinder v. 2.4 [95].

Orthologous protein sequences among all nine species were allocated using OrthoFinder with default settings. Resultant orthogroups were annotated based on the most frequent gene annotation within the orthogroup. Single-copy orthologue sequences were identified and aligned using OrthoFinder, implementing the MAFFT sequence alignment method [96]. The corresponding nucleotide coding sequences (CDS) of these single-copy orthologue protein sequences were extracted from each of the respective genomes, and aligned using the codon-aware sequence alignment method supplied by HyPhy v. 2.5.32 [28,29]. These single-copy orthologue CDS were utilized for our subsequent analyses of selection.

### (e) Gene expansions and contractions

The gene counts generated using OrthoFinder were used to investigate gene family expansions and contractions under a birth–death model using CAFE v. 5.0 [97]. To prepare the input tree for CAFE, the rooted species tree generated by OrthoFinder was converted into an ultrametric tree using the make_ultrametric.py script supplied by OrthoFinder. Gene families with ≥ 100 genes in any one lineage were extracted using a CAFE python script (clade_and_size_filter.py) and analysed separately, because high variance of gene copy number can lead to non-informative parameter estimates. The orthogroup data sets were analysed with CAFE, implementing a Poisson root frequency distribution (-p), a model to account for genome assembly and annotation errors (-e), and a lambda tree (-y) specifying three separate lambda estimations for the termites, cockroaches, and outgroup (following results from CODEML analyses).

### (f) Positive and relaxed selection

We estimated *d*_N_/*d*_S_ values for the aforementioned single-copy orthologues extracted from the nine analysed genomes using CODEML, which is part of the PAML package [25]. We used a fixed tree topology, based on previous phylogenetic estimates (figure 2*b* [18,19,98]), and implemented various *d*_N_/*d*_S_ partitions across the tree in separate analyses. First, we implemented the free-ratio model, which estimates a separate *d*_N_ and *d_S_* value for each branch across the phylogeny. We ran CODEML, implementing the free-ratio model, three times on 1484 single-copy orthologue alignments (seven alignments were removed due to missingness), then extracted the estimated *d*_N_ and *d*_S_ values from the run with the highest likelihood score. To calculate the *d*_N_ and *d*_S_ rates, we scaled these values with branch lengths from chronograms derived from Evangelista *et al*. [19] and Beasley-Hall *et al*. [98].

Second, we implemented two- and three-ratio *d*_N_/*d*_S_ models, whereby specific sets of branches are nominated as foreground branches (i.e. the branches ‘of interest’), and other branches are nominated as background branches. In the two-ratio model, we set the branch to the outgroup (*Laupala kohalensis*) as the background branch, allowing a single *d*_N_/*d*_S_ value to be estimated across Blattodea. In the three-ratio model, we allowed separate *d*_N_/*d*_S_ values to be estimated for the termite clade (excluding the termite stem branch) and the cockroach branches (i.e. the rest of Blattodea). We ran CODEML, implementing the two- and three-ratio models, for all 1484 single-copy orthologue alignments. For each orthologue, we used likelihood-ratio tests to evaluate whether separate values for *d*_N_/*d*_S_ for the termites and other taxa in Blattodea fit the sequence data better than a single *d*_N_/*d*_S_ value for Blattodea.

We investigated positive selection for each gene on different branches using an adaptive branch-site random-effects model (aBSREL) [99], implemented in HyPhy. aBSREL estimates *d*_N_/*d*_S_ values and tests whether a proportion of sites have evolved under positive selection for each user-specified test branch in the tree. First, we analysed the four termite branches (i.e. assigned them as the ‘foreground’ branches) to investigate signatures of positive selection related to the evolution of termites. Second, we analysed a larger subset of branches across the cockroach and termite phylogeny (i.e. 12 branches assigned as ‘foreground’ branches) to investigate broader patterns of positive selection in the group. Resultant *p*-values were corrected for multiple branches being tested using the Holm–Bonferroni method (based on the 5% level of significance). We repeated each analysis using three models accounting for multinucleotide mutations: multiple-hits ‘Double’, multiple hits ‘Double + Triple’, and the standard model. Analyses implementing the best-fitting model for each orthologue were retained.

To test for genes with evidence of relaxed selection, we applied the RELAX method [27], implemented in HyPhy [28,29], to each alignment separately. RELAX fits three *d*_N_/*d*_S_ classes to the phylogeny then tests for relaxed/intensified selection on a user-specified test branch. We ran RELAX for 12 different test branches in separate analyses (figure 1*c*). Resultant *p*-values were corrected for multiple branches being tested using the Holm–Bonferroni method (based on the 5% level of significance). To evaluate whether patterns of relaxed selection were due to errors in our automated sequence alignments, we repeated the RELAX analyses on a subset of audited orthologue alignments. We manually inspected and aligned sequences of 105 orthologues, including 20 randomly selected orthologues that were found to be under relaxed selection in all three termite species, 22 that were found to be under relaxed selection in two termite species, and 65 that yielded no evidence of relaxed selection. We subsequently carried out a RELAX analysis on the realigned orthologues, implementing the standard model for multinucleotide mutations. Furthermore, we ran RELAX for all termite branches together (i.e. multiple test branches were considered in a single analysis) and for all cockroach branches together (a Holm–Bonferroni correction was implemented as detailed above).

### (g) Analysis of differentially expressed genes

We identified and analysed caste-biased and unbiased genes characterized by Harrison *et al*. [6] and Maekawa *et al*. [31] in our single-copy orthologue data set based on the putative annotations. We tested whether there were significantly more or fewer caste-biased orthogroups (and unbiased orthogroups) under relaxed or positive selection compared with the whole data set using chi-squared tests, implemented in the R package ‘stats’ [100].

To investigate the evolutionary history of these caste-biased orthogroups (and unbiased orthogroups), we assessed if they were associated with a higher or lower rate of evolution in cockroaches (see [8]). We did this by estimating total tree lengths for the cockroaches for each single-copy orthologue using IQ-TREE v. 2.2.2 [101]. Additional information on estimating tree lengths can be found in the electronic supplementary material. In addition to the tree-length analysis, we investigated the selection signatures of these caste-biased orthogroups in cockroaches, and compared them with the whole data set using chi-squared tests as above.

### (h) Measuring the ‘genetic load’

To investigate genetic load among the sampled taxa, we analysed amino acid substitutions in BUSCOs from the Insecta lineage data set. These highly conserved genes are expected to be under purifying selection, and most derived alleles are likely to be deleterious [102,103]. Here, we focus on ‘realized load’ and not ‘masked load’, as heterozygosity was not considered [49,51].

For this analysis, we extracted BUSCOs from the Insecta lineage data set in each of the sampled taxa using BUSCO v. 5.4.2. We then polarized the amino acid substitutions of the aligned BUSCOs relative to the corresponding BUSCOs from the cricket outgroup. We analysed the derived amino acid substitutions to infer realized genetic load based on three metrics: (1) the total number of derived substitutions per amino acid; (2) the number of putatively deleterious substitutions per amino acid; and (3) the ratio of putatively deleterious to benign substitutions. We used PROVEAN v. 1.1.5 [50] to classify substitutions as either ‘benign’ (PROVEAN scores > −2.5) or ‘deleterious’ (PROVEAN scores ≤ −2.5) based on how phylogenetically conserved the allele is among a database of homologous protein sequences [104–106]. Additional information on estimating genetic load can be found in the electronic supplementary material.

## Data Availability

Genomes assemblies and annotations for the three Blaberidae species (*Geoscapheus dilatatus*, *Neogeoscapheus hanni* and *Panesthia cribrata*) are available from the Dryad Digital Repository: https://doi.org/10.5061/dryad.sqv9s4n9t [107]. The genome assemblies are also archived on GenBank under BioProject PRJNA1065107 (accession numbers JAZBJU000000000, JAZBJV000000000 and JAZBJW000000000 for *Geoscapheus dilatatus*, *Neogeoscapheus hanni* and *Panesthia cribrata*, respectively), and all associated sequence data are archived on the SRA database (BioSample accessions SAMN39450771, SAMN39450772 and SAMN39450773 for *Geoscapheus dilatatus*, *Neogeoscapheus hanni* and *Panesthia cribrata*, respectively). Additional data are provided in the electronic supplementary material [108].
